# Microflora Disturbance during Progression of Glucose Intolerance and Effect of Sitagliptin: An Animal Study

**DOI:** 10.1155/2016/2093171

**Published:** 2016-08-18

**Authors:** Xinfeng Yan, Bo Feng, Peicheng Li, Zhaosheng Tang, Lin Wang

**Affiliations:** Department of Endocrinology, Shanghai East Hospital, Tongji University School of Medicine, Shanghai 200120, China

## Abstract

*Background*. Emerging evidences have shown a close interplay between obesity, diabetes, and intestinal flora disturbance. Dipeptidyl peptidase-4 inhibitor, exemplified by sitagliptin, is highly efficacious in treating type 2 diabetes (T2DM), yet little is known if sitagliptin exerts beneficial effects on microbiota associated with obesity and T2DM. We evaluated changes of gut microbiota following the induction of obesity and T2DM in a streptozotocin treated high fat/high carbohydrate fed (HF/HC-STZ) rat model and explored the effect of sitagliptin on gut microbiota for HF/HC-STZ rats.* Methods*. Sitagliptin was administered via oral gavage to diabetic rats. Fecal DNA extraction and 454 pyrosequencing based on analysis of 16S rRNA genes was utilized to determine the overall structure of microbiota in fecal DNA samples.* Results*. Results showed that, at the level of phylum, there was higher abundance of Firmicutes and Tenericutes and less abundance of Bacteroidetes in obese rats compared to their lean counterparts. At the level of genus, short-chain fatty acid- (SCFA-) producing bacteria,* Blautia*,* Roseburia*, and* Clostridium*, and probiotics* Lactobacillus*,* Bifidobacterium*, and so forth were identified significantly different from each other among conditions.* Conclusion*. Marked shifts of the gut microbiota structure were observed in the rats during development of glucose intolerance. Intestinal flora changed in the process of glucose intolerance, and treatment of sitagliptin moderately corrected the dysbiosis of microbiota in T2DM.

## 1. Introduction

Type 2 diabetes mellitus (T2DM) is exacting a huge level of patient suffering and social cost worldwide. Recent studies suggested that an altered composition and diversity of gut microbiota could play an important role in the development of metabolic disorders. As the second genome of human, intestinal flora has commensal relationship of mutual benefit with the host. Although not fully understood yet, the gut microbiota is implicated in various aspects of intestinal function integrity including epithelial cell turnover, immune modulation, and gastrointestinal motility. The gut microbiota also regulate energy metabolism such as breaking down dietary toxins and carcinogens, manufacturing micronutrients, fermenting indigestible food substances, facilitating the absorption of certain electrolytes and trace minerals, and governing the growth and differentiation of enterocytes through the production of short-chain fatty acids (SCFA) [1–4]. Studies have shown that obesity promotes the growth of the Firmicutes phylum and reduces the proportion of Bacteroidetes phylum in the gut [5–9]. Implantation of gut flora from obese mice to normal and germfree mice resulted in increased body weight and insulin resistance [9, 10] supporting the notion that the bacterial species from obese gut have metabolically unfavorable properties. The associations between microbiota and obesity, insulin resistance, and diabetes are presumably due to the impaired ability of the microbes to extract energy from the diet [9], altered fatty acid metabolism [11], changes in secretion of gut hormones such as peptide YY (PYY) [12], activation of lipopolysaccharide toll-like receptor-2 [13], and changes in the intestinal barrier integrity [14]. There seems to be an obvious growing interest toward the relationship between gut microbes and T2DM.

Sitagliptin, a dipeptidyl peptidase-4 inhibitor, received approval from the US FDA in 2006 for treatment of type 2 diabetes mellitus. It prevents the enzymatic degradation of glucagon-like peptide 1 (GLP-1) and glucagon-like peptide 2 (GLP-2). GLP-1 appears to increase insulin secretion, decrease glucagon secretion, decrease hepatic gluconeogenesis, improve insulin sensitivity, and delay gastric emptying [15]. GLP-2 appears to be an intestinal specific growth factor, promoting the growth of intestinal mucosa, repairing damaged intestinal epithelium, and improving the intestinal mucosa barrier integrity [16, 17]. Since sitagliptin affects the metabolism of GLP-1 and GLP-2 secreted from enteroendocrine, we wonder whether sitagliptin regulates intestinal flora.

Among available methodologies to study the microbial ecology of complex bacterial communities, the so-called metagenomics approach is considered to be the “golden standard” [18, 19]. In the current study, we analyzed the structure of intestinal flora in a HF/HC progressive glucose intolerance rat model with or without sitagliptin treatment by 454 pyrosequencing; we tried to explore whether and to what extent sitagliptin regulated microbiota.

## 2. Materials and Methods

### 2.1. Drug and Diets

The HF/HC diet (containing 19.8 g fat, 44.6 g carbohydrate, and 22.3 g protein per 100 g, and 40 kcal% fat, 40 kcal% carbohydrate, and 20 kcal% protein by energy) was purchased from Shanghai Laboratory Animal Center, Chinese Academy of Science (SLACCAS), Laboratory Animal Co. Ltd. (Shanghai, China). The streptozotocin (STZ) was purchased from Sigma, USA. Sitagliptin was a kind gift from MSD China.

### 2.2. Animal Experiments

Fifteen four-week-old male Sprague-Dawley (SD) rats (pathogen-free grade, average body weight 110 g) were purchased from SLACCAS Laboratory Animal (Shanghai, China). All rats were acclimatized in our laboratory for 7 days before the initiation of the experiment. After fasting for 12 hours, fresh stool samples were collected by stimulating the anus and immediately stored at −80°C for subsequent analysis. Then the rats were fed with HF/HC diet for 4 weeks, and stool samples were collected for the second time. After fasting for 12 hours, an OGTT test was performed to assess the insulin resistance. The rats were receiving 50% D-glucose solution by gavage at 2 g/kg body weight, and blood glucose levels were measured at 0, 30, 60, 90, and 120 minutes following the glucose challenge using blood samples collected by tail snipping. The day after OGTT test, the HF/HC fed (4 weeks on the diet) rats were injected intraperitoneally with STZ (30 mg/kg body weight) to induce diabetes. The animals were fed continuously on the HF/HC diet throughout the rest of the study. Ten rats developed diabetes with fasting blood glucose (FBG) >11.1 mmol/L at 2 weeks after STZ treatment [20, 21]. Two rats died and 3 rats failed to develop diabetes. Stool samples were collected from the rest of the 10 rats for the third time 4 weeks after the induction of T2DM. Thereafter, sitagliptin (10 mg/kg body weight, oral gavage, once a day) was administered to all 10 diabetic rats for 12 weeks, followed by another stool sample collection (Figure S1 in Supplementary Material available online at http://dx.doi.org/10.1155/2016/2093171). Body weight was weighed and blood glucose was checked weekly with Bayer glucometer during the study.

All animal experimental protocols were approved by the Animal Ethics Committee of Animal Center of East Hospital, Tongji University, and all the animal experiments were carried out in strict accordance with the Guidelines for Care and Use of Laboratory Animals of the Animal Ethics Committee of Animal Center of East Hospital, Tongji University. All efforts were made to minimize animal suffering.

### 2.3. Fecal DNA Extraction and 454 Pyrophosphate Sequencing

Microbial DNA was extracted from stool samples using the E.Z.N.A. Stool DNA Kit (Omega Bio-tek, Norcross, GA, USA) according to manufacturer's instructions. The V1–V3 regions of the bacteria 16S ribosomal DNA gene were amplified by polymerase chain reaction (95°C for 2 min, followed by 25 cycles at 95°C for 30 s, 55°C for 30 s, and 72°C for 30 s and a final extension at 72°C for 5 min) using primers 27F 5′-(CCTATCCCCTGTGTGCCTTGGCAGTCGACT-3′5′-AGAGTTTGATCCTGGCTCAG)-3′ and 533R 5′-(CCATCTCATCCCTGCGTGTCTCCGACGACT-3′-MID tgas-5′-TTACCGCGGCTGCTGGCAC)-3′. PCR reactions were performed in a 20 *μ*L mixture containing 4 *μ*L of 5x FastPfu Buffer, 2 *μ*L of 2.5 mM dNTPs, 0.8 *μ*L of each primer (5 *μ*M), 0.4 *μ*L of FastPfu Polymerase, and 10 ng of template DNA. After purification with the AxyPrep DNA Gel Extraction Kit (Axygen Biosciences, Union City, CA, USA) and quantification using QuantiFluor-ST (Promega, US), the amplified mixture was used for pyrosequencing on a Roche 454 GS FLX+ Titanium platform (Roche 454 Life Sciences, Branford, CT, USA) according to standard protocols at Majorbio Bio-Pharm Technology Co., Ltd., Shanghai, China. The raw reads were deposited into the NCBI Sequence Read Archive (SRA) database (Accession Number: SRP056522).

In total, 323,039 valid sequences were obtained from all 40 samples, with an average length of 419 bp per sequence. The resulting sequences were processed using QIIME (version 1.17). After removing sequences with average quality score <20 over a 50 bp sliding window, sequences shorter than 200 bp, sequences with homopolymers longer than six nucleotides, and sequences with ambiguous base calls or incorrect primer sequences, a total of 241,879 high-quality sequences were produced with an average length of 430 bp per sequence. Operational Taxonomic Units (OTUs) were clustered with 97% similarity cutoff using UPARSE (version 7.1 http://drive5.com/uparse/) and chimeric sequences were identified and removed using UCHIME. The phylogenetic affiliation of each 16S rDNA gene sequence was analyzed by RDP Classifier (Michigan State University, http://rdp.cme.msu.edu/) against the SILVA (SSU115) 16S rDNA database using confidence threshold of 70% [22].

### 2.4. Statistical Analysis

Comparisons of diversity estimators and the metabolic indices between conditions were analyzed by the one-way repeated measures ANOVA using SPSS version 13.0 for Windows. OTUs that reached 97% similarity level were used for diversity (Shannon), rarefaction curve and Shannon-Wiener curve analysis by using Mothur (version 1.30.1) [23]. Heatmap figure was generated using R packages gplots [24] at genus level. A differentially abundance feature was analyzed based on multiple hypotheses testing rare frequency data and false discovery rate (FDR) analysis using Metastat analysis [25]. Principal coordinates analysis (PCoA) was performed based on unweighted UniFrac distance. In addition, linear discriminant analysis effect size (LEfSe) was performed first based on nonparametric factorial Kruskal-Wallis (KW) sum-rank test and then we performed the linear discriminant analysis following the Wilcoxon Signed-Rank test to assess effect size of each differentially abundant taxon or OTU.

## 3. Results

### 3.1. Body Weight and Blood Glucose

As shown in Table 1, HF/HC diet resulted in significant increase of body weight (398.92 ± 23.06 g in obesity condition versus 155.06 ± 6.4 g in normal condition, *P* < 0.01). Furthermore, STZ injection resulted in dramatic increase of blood glucose level in diabetes condition as compared to normal or obesity condition (*P* < 0.01). As expected, sitagliptin resulted in a significant reduction of blood glucose (*P* < 0.05) while having no significant impacts on body weight (*P* = ns).

### 3.2. OGTT Test

The fasting blood glucose of obese rats was significantly higher than that in normal control condition (4.30 ± 0.37 mmol/L versus 3.83 ± 0.25 mmol/L, *P* < 0.01), and blood glucose level at 120 min was 8.92 ± 1.23 mmol/L which should be considered IGT by definition [26]. Data are expressed as mean ± SD.

### 3.3. Characteristics of 454 Pyrosequencing Results

**Figure 1:**
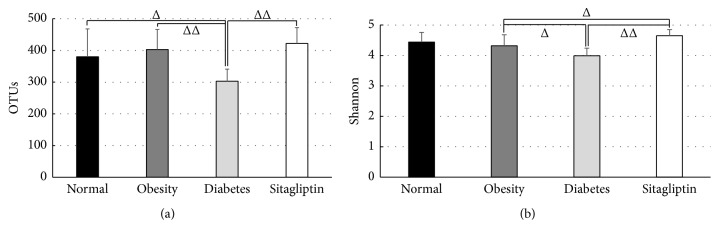
Pyrosequencing data summary: (a) number of OTUs, (b) Shannon (diversity estimator). All data were calculated at 3% distance. ^Δ^ *P* < 0.05, ^ΔΔ^ *P* < 0.01; data are expressed as mean ± SD.

A total of 241,879 high-quality sequences of 40 samples were produced in this study, with an average of 6047 sequences per sample. A brief summary and the estimators of OTUs and diversity (Shannon) are shown in Figure 1, and detailed characteristics of each sample are listed in Table S1. There were statistically significant differences of Shannon indexes between obese condition and sitagliptin-treated condition (4.32 ± 0.36 versus 4.65 ± 0.20, *P* < 0.05) and between diabetic condition and sitagliptin-treated condition (3.99 ± 0.25 versus 4.65 ± 0.20, *P* < 0.01), suggesting significant higher diversity found in sitagliptin condition compared to obese or diabetic condition.

The rarefaction curves of all four conditions did not level off at the sequencing depth of 6,000 (Figure S2). Therefore, this sequencing depth was not sufficient to cover the whole bacterial diversity. Thus, it deserves further sequencing to detect more bacterial species in the rat feces. However, when the Shannon curves tended to be smooth, the sequencing data quantity became dramatically bigger, which reflected the vast majority of microbes in the sample (Figure S3).

### 3.4. Microbial Structures Differed Significantly among Conditions

**Figure 2:**
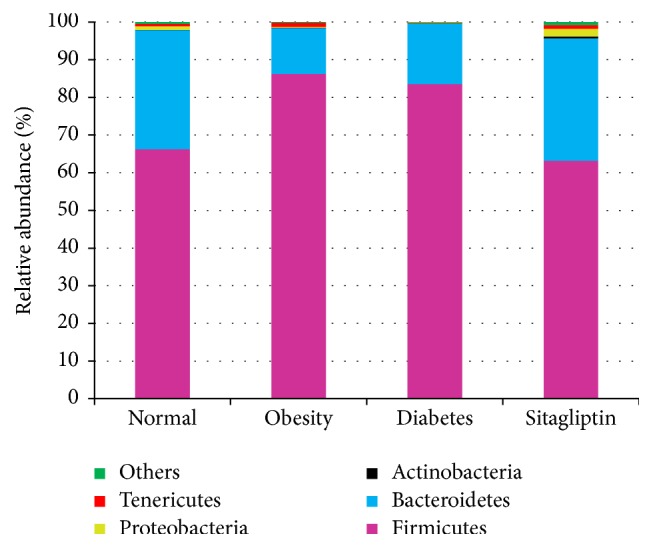
Relative abundance of different bacterial phyla in microbiota of four conditions.

There were significant differences at the phylum level in rat fecal microbiota during progression of glucose intolerance and after treatment with sitagliptin.  Figure 2 demonstrated that Bacteroidetes and Firmicutes accounted for the majority of microflora (>90%) in each condition. The relative abundance of Firmicutes in obesity condition was significantly increased compared to that in the normal condition (86.28% versus 66.24%, *P* < 0.01), while the relative abundance of Bacteroidetes decreased substantially (12.16% versus 31.64%, *P* < 0.01). There was no significant difference between obesity and diabetes at phylum level. The relative abundance of Firmicutes in the sitagliptin condition was significantly less than that in the diabetic condition (63.19% versus 83.56%, *P* < 0.01). In contrast, the relative abundance of Bacteroidetes increased dramatically (32.46% versus 16.06%, *P* < 0.01). It is noteworthy that the relative abundance of Tenericutes in the obese condition significantly increased compared to the normal condition (1.15% versus 0.61%, *P* < 0.05) but decreased dramatically after inducing diabetes (1.15% versus 0.09%, *P* < 0.01) and then increased largely after sitagliptin treatment as compared to the diabetic condition (0.96% versus 0.09%, *P* < 0.01). Similar changes were observed at the phylum of Proteobacteria.

**Figure 3:**
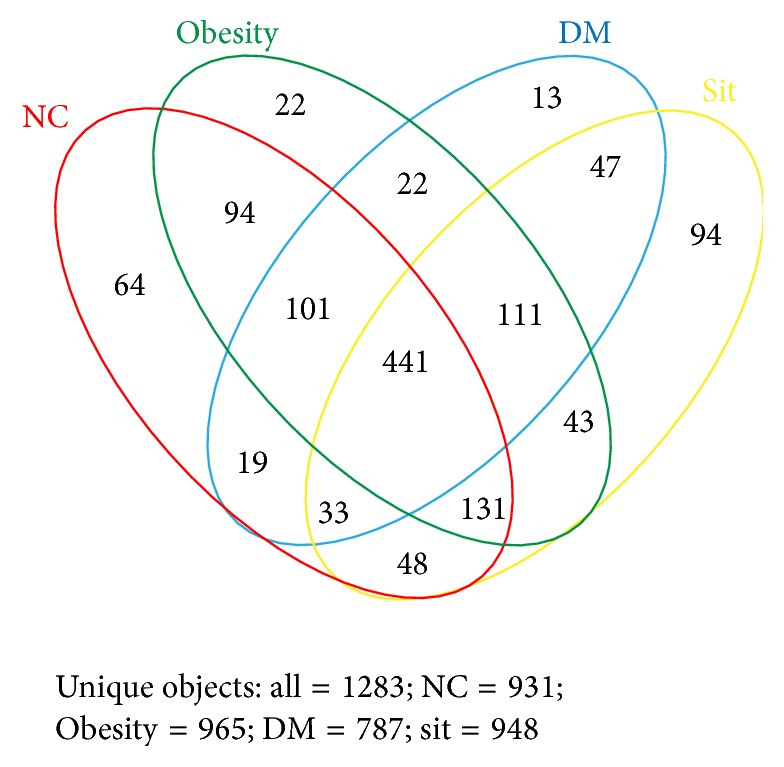
Shared OTU analysis of the different conditions. Venn diagram showing the unique and shared OTUs (3% distance level) in the different conditions.

Furthermore, we explored the similarities and distinctions of species distribution in all conditions. As shown in Figure 3, we found that there were 441 species shared in the four conditions, accounting for around half of the OTUs in each condition. It is noteworthy that about one-third of the species only found in normal (64 OTUs) or obesity (22 OTUs) condition belonged to Lachnospiraceae, which is capable of fermenting complex carbohydrates to SCFAs playing an important role in maintaining intestinal homeostasis [27, 28]. Interestingly, one-third of the total species only found in sitagliptin condition (94 OTUs) belonged to Ruminococcaceae, which is a major utilizer of plant polysaccharides [29, 30].

In addition, Metastat analysis showed significant differences of relative abundance at the level of genus or family among conditions (Table 2 and Table S2). And the result at level of genus was marked in the Heatmap plot (Figure S4) which showed the significant differences of Blautia, Roseburia, Clostridium, and so on, among four conditions.

**Figure 4:**
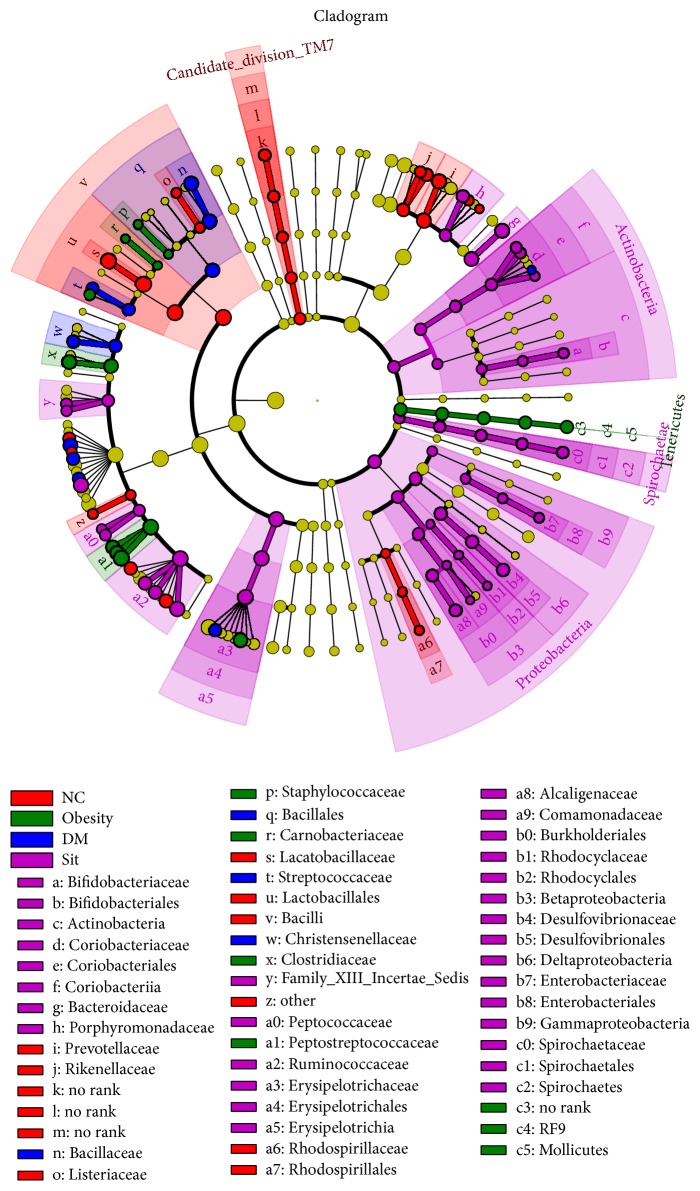
Taxonomic representation of statistically and biologically consistent differences among condition normal, obesity, diabetes, and sitagliptin. Differences are represented by the color of the most abundant class (red indicating normal condition, green obesity condition, blue diabetes condition, purple sitagliptin condition, and yellow nonsignificance). The diameter of each circle is proportional to the taxon's abundance.

There were significant variations in the composition of intestinal bacteria species among four study conditions. A cladogram representation of the microbiota structure of four conditions and their predominant bacteria performed by LEfSe and the greatest differences in taxa among four communities are shown in Figure 4 and Figure S5.

**Figure 5:**
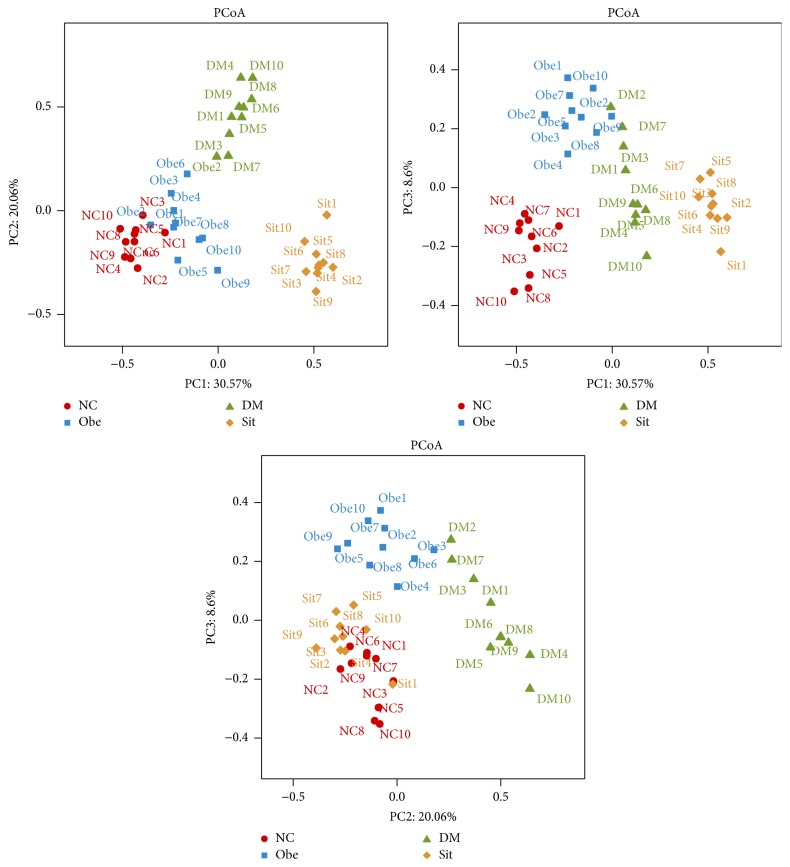
PCA plots based on unweighted Unifrac metrics. Principal components (PCs) 1, 2, and 3 explained 30.57%, 20.06%, and 8.6%. Each symbol represents a sample.

To compare the overall microbiota structures in all conditions, the unweighted Unifrac distance matrix was calculated based on the OTUs of each sample. The PCA result revealed a significant difference in gut bacterial structure among all conditions. The three principal component scores accounted for 30.57%, 20.06%, and 8.6% of total variations, respectively (Figure 5).

## 4. Discussion

In the study, we explored the changes of rat intestinal microbiota during progression of glucose intolerance and the effect of sitagliptin on microbiota. We found significantly more Firmicutes and less Bacteroidetes in obese rats compared to their lean counterparts, similar to previous studies [31] despite conflicting data showing no differences of Bacteroidetes and Firmicutes between obese and lean subjects [32–34]. The increased Firmicutes in obese mice may be related to the genes encoding enzymes that break down polysaccharides which cannot be digested by the host, increasing the production of monosaccharides and short-chain fatty acids (SCFA) and the conversion of these SCFA to triglycerides in the liver. The binding of SCFA to two G-protein-coupled receptors (GPR41 and GPR43) induces peptide YY secretion, which suppresses gut motility, retards intestinal transit, and subsequently results in the increase of nutrient uptake and deposition [35]. Interestingly, we also found that hydrogen-producing Prevotellaceae decreased significantly in the obese condition. Given the fact that hydrogen inhibits digestion, the obese rats seemed likely to absorb more calories from similar energy intake.

Another interesting finding was the higher abundance of the phylum Tenericutes (class Mollicutes) in obese rats compared to their lean counterparts. Certain species of Mollicutes bloom have been proved to evolve the capacity to import certain types of carbohydrates common in westernized diet for both mice and human being (e.g., glucose, fructose, and sucrose) and to metabolize these imported sugars to SCFA which could be readily absorbed by the host [36]. Sitagliptin did restore the structure of gut microbe at the level of phylum, similar to the lean control condition without significant effects on body weight.

Unweighted Unifrac PCA analysis confirmed above results in the respect of overall microbial structures. The intestinal microbiota of the four conditions of rats were structurally separated from each other in the three principal components.

Various mechanisms have been proposed to explain the influence of microbiota on insulin resistance and T2DM including metabolic endotoxemia, modifications in the secretion of the incretins, and butyrate production. Decrease of SCFA-producing bacteria has been commonly observed in metabolic diseases including T2DM [37] and even colorectal cancer [38]. SCFA-producing bacteria have been previously shown to benefit the host through protecting the mucosa from damage induced by pathogens, supplying colonocyte nutrients, mitigating inflammation, and so forth [39, 40]. Recent studies in mice have shown that an increase in colonic production of short-chain fatty acids triggers intestinal gluconeogenesis (IGN) via complementary mechanisms. Butyrate activates IGN gene expression through a cAMP-dependent mechanism in enterocytes, whereas propionate, itself a substrate of IGN, activates IGN gene expression via the portal nervous system and the fatty acid receptor FFAR3. In rodents, the result of increased IGN is beneficial to glucose and energy homeostasis with reductions in hepatic glucose production, appetite, and body weight [41]. Belonging to either* Clostridium*,* Eubacterium,* or* Fusobacterium,* the butyrate-producing bacteria mainly exist in cecum and colon.* Fusobacterium prausnitzii* and* Roseburia intestinalis* are predominant species in butyric acid producing bacteria in human and animal intestine [42]. At the level of genus, our data suggested that the relative abundance of butyrate-producing* Clostridium* and* Roseburia* in the diabetic rats decreased significantly compared to the lean and obese conditions. Moreover, we also observed that the SCFA-producing bacteria* Blautia* increased significantly in the diabetic condition compared to the obese condition. Becker et al. demonstrated that the addition of* Clostridium butyricum* as a second butyrate-producing bacterium to SIHUMI (a simplified human intestinal microbiota:* Anaerostipes caccae, Bacteroides thetaiotaomicron, Bifidobacterium longum, Blautia producta, Clostridium ramosum, Escherichia coli, *and* Lactobacillus plantarum*) led to an increase of butyrate by 56% but decrease of* Bifidobacterium longum*,* Blautia producta* and* E. coli*, indicating that either of these organisms competes with* Clostridium butyricum* for the same substrates or* Clostridium butyricum* forms inhibitory substances [43]. Thus it seems reasonable to suppose that changes of* Clostridium*,* Roseburia,* and* Blautia* show an opposite trend. In other words, the increase of SCFA-producing bacteria like* Blautia* leads to decrease of butyrate-producing bacteria like* Clostridium *and* Roseburia*. In the current study, the structure of SCFA-producing bacteria seemed to be abnormal in diabetic rats. After sitagliptin treatment,* Roseburia *increased and* Blautia* decreased while* Clostridium* showed no change.

Probiotics such as* Lactobacillus* and* Bifidobacterium* normally reside in the intestinal tract and regulate gut microflora and mucosal immunity. Recent data also suggest that microflora, especially probiotics, exert beneficial effects on gastrointestinal discomforts such as diarrhea, abdominal pain, and bloating [44].* Lactobacillus* produces lactic acid, CO_2_, acetic acid, and/or ethanol which may contribute to a more acidic environment through homo- or heterofermentative metabolism [45, 46]. Our data showed that* Lactobacillus* and* Bifidobacterium* decreased in the diabetic condition. However, sitagliptin made* Bifidobacterium* increased with no significant effect on* Lactobacillus*, which seemed to be consistent with the notion we discussed above.

Marked changes in microbiota composition after metformin treatment were observed in high-fat diet- (HFD-) fed conditions, especially* Akkermansia* belonging to the Verrucomicrobia phylum showed the most conspicuous changes [47], suggesting a possible interaction between HFD, metformin, and intestinal microbiota. Forslund et al. found significant increase of* Escherichia *spp. and decrease of* Intestinibacter *spp. in human gut microbiome in metformin-treated T2DM; especially the latter is resistant to oxidative stress and able to degrade fucose, indicative of an indirect involvement in mucus degradation [41]. Interestingly, there are consistent data from numerous studies showing that sitagliptin results in reduced gastrointestinal discomfort when combined with metformin, a classic antihyperglycemia agent also known for its gastrointestinal side effects. For instance, Reasner et al. showed that sitagliptin and metformin fixed dose combination resulted in a significant reduction on abdominal pain and diarrhea compared to metformin monotherapy [48]. In addition, a 104-week clinical study (Harmony) also showed that diarrhea incidence was reduced in sitagliptin plus metformin group as compared to metformin monotherapy group [49]. Though the underlying mechanism of effect of metformin and sitagliptin on intestinal flora remains unclear, our results seem to provide a possible explanation arguing that this effect could be related to favorable changes of microflora after sitagliptin treatment.

We supposed that the effect of sitagliptin on microbiota was related to GLP-2, which helps improve the intestinal mucosa barrier integrity. Besides, recent studies found that DPP-4 may act as the protease activated receptor 2 (PAR2) agonists involved in the pathophysiology of PAR2, leading to proliferation and inflammation in smooth muscle cells. Thus DPP-4 inhibitor can relieve edema of intestinal wall and alleviate the intestinal inflammation [50, 51]. Integrated intestinal mucosa barrier and improved intestinal environment may be not fit for bacteria related with obesity and diabetes or opportunistic pathogen to colonize.

In summary, our data highlights the potential beneficial effect of sitagliptin on gut microbe of diabetic or obese animals, which constituted a novel, exciting observation calling for further investigation on numerous involved microbiota species. These data, when combined with the results of future clinical studies, may facilitate the development or optimization of novel microbiota based T2DM interventional strategies.

## 5. Conclusions

Taken together, our data suggest that the structure of gut flora, especially SCFA-producing bacteria, seems to be abnormal in a progressive glucose intolerance rat model. Treatment with sitagliptin improved gut dysbiosis moderately. To the best of our knowledge, our study seems to be the first report showing that a DPP-4 inhibitor may exert positive regulation on gut flora dysregulated during progression of glucose intolerance and obesity in an animal model. Future research will be directed to elucidate whether the favorable changes of microflora contribute to better metabolic control and reduced gastrointestinal side effects of DPP4 inhibitor when combined with metformin.

## Supplementary Material

Figure S1 Process of animal experiment: The SD rats were induced IGT and T2DM by high-fat-high-sugar chow and low dose streptozocin injection. Diabetic rats were then treated with sitagliptin. Feces were collected at four points in the process, representing normal control, obesity, diabetes and sitagliptin-treated condition respectively.

## Figures and Tables

**Table 1:** Body weight and blood glucose changes during progression of glucose intolerance and after Sitagliptin treatment. Data are expressed as mean ± SD (compared with group obesity: ^○^ *P* < 0.01; compared with group diabetes: ^∆^ *P* < 0.01, ^▲^ *P* < 0.05).

Conditions	Body weight (g)	Blood glucose (mmol/L)
Normal	155.06 ± 6.4^○^	3.83 ± 0.25^○^
Obesity	398.92 ± 23.06	4.30 ± 0.37^∆^
Diabetes	416.83 ± 35.64	18.81 ± 2.55^○^
Sitagliptin	432.67 ± 58.10	15.54 ± 1.39^○▲^

**Table 2:** Statistical analysis was performed by Wilcoxon Signed-Rank test. Data of normal, obesity, diabetes, and sitagliptin are relative abundance (percentage) of all sequences in each group (*P* ^#^ group obesity versus normal; *P* ^*∗*^ group diabetes versus obesity; *P* ^∆^ group diabetes versus sitagliptin).

Taxonomy	Normal (%)	Obesity (%)	Diabetes (%)	Sitagliptin (%)	*P* ^#^	*P* ^*∗*^	*P* ^∆^
*Bacteroides* (genus)	1.4	0.2	0.6	5.4	0.013	0.022	0.005
Prevotellaceae (family)	9.9	1.8	6.8	9.2	0.005	0.001	
*Blautia* (genus)	0.2	0.8	7.1	0.3	0.012	0.005	0.005
*Roseburia* (genus)	0.7	0.9	0.3	0.5		0.042	
*Ruminococcus* (genus)	2.5	1.4	0.6	1.9		0.025	0.007
*Clostridium* (genus)	1.3	4.6	0.8	0.7	0.014	0.013	
*Lactobacillus* (genus)	22.3	22.5	13.0	6.7			0.028
*Bacillus* (genus)	4.6	4.4	6.0	0			0.005
*Bifidobacterium* (genus)	0.024	0.005	0.002	0.085			0.007
*Parabacteroides* (genus)	0.2	0.02	0.1	0.6	0.008	0.024	0.005

## References

[1] Ouwehand A., Isolauri E., Salminen S. (2002). The role of the intestinal microflora for the development of the immune system in early childhood. *European Journal of Nutrition*.

[2] Stappenbeck T. S., Hooper L. V., Gordon J. I. (2002). Developmental regulation of intestinal angiogenesis by indigenous microbes via Paneth cells. *Proceedings of the National Academy of Sciences of the United States of America*.

[3] Klaenhammer T., Altermann E., Arigoni F. (2002). Discovering lactic acid bacteria by genomics. *Antonie van Leeuwenhoek*.

[4] Makarova K., Slesarevb A., Wolf Y. (2006). Comparative genomics of the lactic acid bacteria. *Proceedings of the National Academy of Sciences of the United States of America*.

[5] Esteve E., Ricart W., Fernández-Real J.-M. (2011). Gut microbiota interactions with obesity, insulin resistance and type 2 diabetes: did gut microbiote co-evolve with insulin resistance?. *Current Opinion in Clinical Nutrition and Metabolic Care*.

[6] Backhed F., Ding H., Wang T. (2004). The gut microbiota as an environmental factor that regulates fat storage. *Proceedings of the National Academy of Sciences of the United States of America*.

[7] Bajzer M., Seeley R. J. (2006). Physiology: obesity and gut flora. *Nature*.

[8] Turnbaugh P. J., Hamady M., Yatsunenko T. (2009). A core gut microbiome in obese and lean twins. *Nature*.

[9] Turnbaugh P. J., Ley R. E., Mahowald M. A., Magrini V., Mardis E. R., Gordon J. I. (2006). An obesity-associated gut microbiome with increased capacity for energy harvest. *Nature*.

[10] Ley R. E., Bäckhed F., Turnbaugh P. (2005). Obesity alters gut microbial ecology. *Proceedings of the National Academy of Sciences of the United States of America*.

[11] Velagapudi V. R., Hezaveh R., Reigstad C. S. (2010). The gut microbiota modulates host energy and lipid metabolism in mice. *Journal of Lipid Research*.

[12] Diamant M., Blaak E. E., de Vos W. M. (2011). Do nutrient-gut-microbiota interactions play a role in human obesity, insulin resistance and type 2 diabetes?. *Obesity Reviews*.

[13] Caricilli A. M., Picardi P. K., de Abreu L. L. (2011). Gut microbiota is a key modulator of insulin resistance in TLR 2 knockout mice. *PLoS Biology*.

[14] Cani P. D., Possemiers S., Van De Wiele T. (2009). Changes in gut microbiota control inflammation in obese mice through a mechanism involving GLP-2-driven improvement of gut permeability. *Gut*.

[15] Drucker D. J. (2003). Enhancing incretin action for the treatment of type 2 diabetes. *Diabetes Care*.

[16] Drucker D. J., Yusta B. (2014). Physiology and pharmacology of the enteroendocrine hormone glucagon-like peptide-2. *Annual Review of Physiology*.

[17] Marathe C. S., Rayner C. K., Jones K. L., Horowitz M. (2013). Glucagon-like peptides 1 and 2 in health and disease: a review. *Peptides*.

[18] Eckburg P. B., Bik E. M., Bernstein C. N. (2005). Microbiology: diversity of the human intestinal microbial flora. *Science*.

[19] Gill S. R., Pop M., DeBoy R. T. (2006). Metagenomic analysis of the human distal gut microbiome. *Science*.

[20] Zhou Y. S., Gao Y., Li B. (2005). A rat model of type 2 diabetes mellitus induced by high fat chow and low dose streptozocin injection. *Acta Laboratorium Animals Scientia Sinica*.

[21] Jialin Y., Guo L., Youpin L. (2003). Establishing a rat model similar to the adult patient of the general type 2 diabetes by long-term fat-enriched fed and lower dose of streptozocin-treated rats. *Acta Laboratorium Animals Scientia Sinica*.

[22] Amato K. R., Yeoman C. J., Kent A. (2013). Habitat degradation impacts black howler monkey (Alouatta pigra) gastrointestinal microbiomes. *ISME Journal*.

[23] Schloss P. D., Westcott S. L., Ryabin T. (2009). Introducing mothur: open-source, platform-independent, community-supported software for describing and comparing microbial communities. *Applied and Environmental Microbiology*.

[24] Warnes G. R., Bolker B., Bonebakker L. (2015). *gplots: Various R Programming Tools for Plotting Data*.

[25] White J. R., Nagarajan N., Pop M. (2009). Statistical methods for detecting differentially abundant features in clinical metagenomic samples. *PLoS Computational Biology*.

[26] Wu Y. L., Zhao B.-Z., Xi G.-X., Gao C.-H. (2007). An experimental study of impaired glucose tolerance in Wistar rats. *Chinese Remedies & Clinics*.

[27] Cotta M., Forster R. (2006). The family lachnospiraceae, including the genera butyrivibrio, lachnospira and roseburia. *The Prokaryotes: Volume 4: Bacteria: Firmicutes, Cyanobacteria*.

[28] Wong J. M. W., de Souza R., Kendall C. W. C., Emam A., Jenkins D. J. A. (2006). Colonic health: fermentation and short chain fatty acids. *Journal of Clinical Gastroenterology*.

[29] Flint H. J., Scott K. P., Duncan S. H., Louis P., Forano E. (2012). Microbial degradation of complex carbohydrates in the gut. *Gut Microbes*.

[30] Ze X., Duncan S. H., Louis P., Flint H. J. (2012). Ruminococcus bromii is a keystone species for the degradation of resistant starch in the human colon. *ISME Journal*.

[31] Ley R. E. (2010). Obesity and the human microbiome. *Current Opinion in Gastroenterology*.

[32] Duncan S. H., Lobley G. E., Holtrop G. (2008). Human colonic microbiota associated with diet, obesity and weight loss. *International Journal of Obesity*.

[33] Mai V., McCrary Q. M., Sinha R., Glei M. (2009). Associations between dietary habits and body mass index with gut microbiota composition and fecal water genotoxicity: an observational study in African American and Caucasian American volunteers. *Nutrition Journal*.

[34] Jumpertz R., Le D. S., Turnbaugh P. J. (2011). Energy-balance studies reveal associations between gut microbes, caloric load, and nutrient absorption in humans. *The American Journal of Clinical Nutrition*.

[35] Erejuwa O. O., Sulaiman S. A., Ab Wahab M. S. (2014). Modulation of gut microbiota in the management of metabolic disorders: The prospects and challenges. *International Journal of Molecular Sciences*.

[36] Turnbaugh P. J., Bäckhed F., Fulton L., Gordon J. I. (2008). Diet-induced obesity is linked to marked but reversible alterations in the mouse distal gut microbiome. *Cell Host and Microbe*.

[37] Qin J., Wang J., Li Y. (2012). A metagenome-wide association study of gut microbiota in type 2 diabetes. *Nature*.

[38] Wang T., Cai G., Qiu Y. (2012). Structural segregation of gut microbiota between colorectal cancer patients and healthy volunteers. *The ISME Journal*.

[39] Maslowski K. M., Vieira A. T., Ng A. (2009). Regulation of inflammatory responses by gut microbiota and chemoattractant receptor GPR43. *Nature*.

[40] De Filippo C., Cavalieri D., Di Paola M. (2010). Impact of diet in shaping gut microbiota revealed by a comparative study in children from Europe and rural Africa. *Proceedings of the National Academy of Sciences of the United States of America*.

[41] Forslund K., Hildebrand F., Nielsen T. (2015). Disentangling type 2 diabetes and metformin treatment signatures in the human gut microbiota. *Nature*.

[42] Hold G. L., Schwiertz A., Aminov R. I., Blaut M., Flint H. J. (2003). Oligonucleotide probes that detect quantitatively significant groups of butyrate-producing bacteria in human feces. *Applied and Environmental Microbiology*.

[43] Becker N., Kunath J., Loh G., Blaut M. (2011). Human intestinal microbiota: characterization of a simplified and stable gnotobiotic rat model. *Gut Microbes*.

[44] Nobaek S., Johansson M.-L., Molin G., Ahrné S., Jeppsson B. (2000). Alteration of intestinal microflora is associated with reduction in abdominal bloating and pain in patients with irritable bowel syndrome. *The American Journal of Gastroenterology*.

[45] Macfarlane G. T., Macfarlane S. (1997). Human colonic microbiota: ecology, physiology and metabolic potential of intestinal bacteria. *Scandinavian Journal of Gastroenterology*.

[46] Zoetendal E. G., Akkermans A. D. L., Akkermans-van Vliet W. M., De Visser J. A. G. M., De Vos W. M. (2001). The host genotype affects the bacterial community in the human gastrointestinal tract. *Microbial Ecology in Health and Disease*.

[47] Shin N.-R., Lee J.-C., Lee H.-Y. (2014). An increase in the *Akkermansia* spp. population induced by metformin treatment improves glucose homeostasis in diet-induced obese mice. *Gut*.

[48] Reasner C., Olansky L., Seck T. L. (2011). The effect of initial therapy with the fixed-dose combination of sitagliptin and metformin compared with metformin monotherapy in patients with type 2 diabetes mellitus. *Diabetes, Obesity and Metabolism*.

[49] Ahrén B., Johnson S. L., Stewart M. (2014). HARMONY 3: 104-week randomized, double-blind, placebo- and active-controlled trial assessing the efficacy and safety of albiglutide compared with placebo, sitagliptin, and glimepiride in patients with type 2 diabetes taking metformin. *Diabetes Care*.

[50] Enjoji S., Ohama T., Sato K. (2014). Regulation of epithelial cell tight junctions by protease-activated receptor 2. *The Journal of Veterinary Medical Science*.

[51] Wronkowitz N., Görgens S. W., Romacho T. (2014). Soluble DPP4 induces inflammation and proliferation of human smooth muscle cells via protease-activated receptor 2. *Biochimica et Biophysica Acta*.

