# Supplementary material for: Microflora Disturbance during Progression of Glucose Intolerance and Effect of Sitagliptin: An Animal Study
Source: J Diabetes Res. 2016 Aug 18;2016:2093171. doi: 10.1155/2016/2093171 (PMC5007364; doi:10.1155/2016/2093171)

Title: Microflora disturbance during progression of glucose intolerance and effect of Sitagliptin: an animal study

Author: Xinfeng Yan, Bo Feng, Peicheng Li, Zhaosheng Tang, Lin Wang


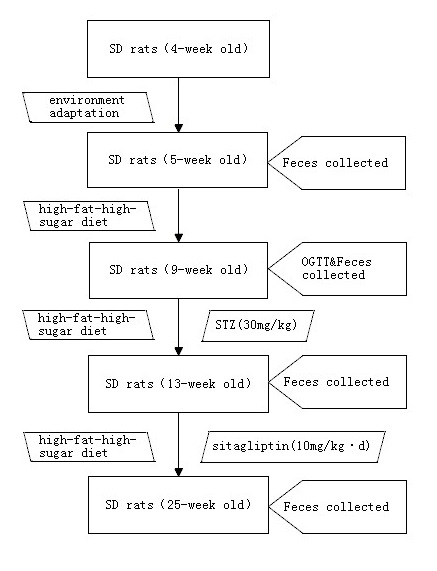

Supplement: Supplementary file 1 — Figure S1 Process of animal experiment: The SD rats were induced IGT and T2DM by high-fat-high-sugar chow and low dose streptozocin injection. Diabetic rats were then treated with sitagliptin. Feces were collected at four points in the process, representing normal control, obesity, diabetes and sitagliptin-treated condition respectively. [file 2093171.f1.zip › Fig.S1-flow chart.docx]
