# Supplementary material for: Microflora Disturbance during Progression of Glucose Intolerance and Effect of Sitagliptin: An Animal Study
Source: J Diabetes Res. 2016 Aug 18;2016:2093171. doi: 10.1155/2016/2093171 (PMC5007364; doi:10.1155/2016/2093171)
Supplement: Supplementary file 1 — Figure S1 Process of animal experiment: The SD rats were induced IGT and T2DM by high-fat-high-sugar chow and low dose streptozocin injection. Diabetic rats were then treated with sitagliptin. Feces were collected at four points in the process, representing normal control, obesity, diabetes and sitagliptin-treated condition respectively. [file 2093171.f1.zip › Fig.S2-Rarefaction Curve.docx]

500

NC6

NC2 NC4

NC1

400

NC9

NC7

NC3

300

NC5

Rarefaction Measure:rarefaction

NC10

NC8

200

100

0

0 2000 4000 6000 8000

Number of Reads Sampled

label: 0.97

500

Obe9

Obe1

Obe7

400

Obe5 Obe10

Obe6 Obe2

**Rarefaction Measure:rarefaction**

Obe8

Obe3

300

Obe4

200

100

0

0 1000 2000 3000 4000 5000 6000 7000

# Number of Reads Sampled

label: 0.97

400

DM7

DM3

DM6 DM8

300

DM2

DM5

DM9

DM4

DM10

DM1

Rarefaction Measure:rarefaction

200

100

0

0 2000 4000 6000 8000

Number of Reads Sampled

label: 0.97

500

Sit7

400

Sit4

Sit8 Sit9

Sit5 Sit2

Sit3 Sit6

Sit10

300

Sit1

**Rarefaction Measure:rarefaction**

200

100

0

0 1000 2000 3000 4000 5000 6000

# Number of Reads Sampled

label: 0.97
