# Supplementary material for: Microflora Disturbance during Progression of Glucose Intolerance and Effect of Sitagliptin: An Animal Study
Source: J Diabetes Res. 2016 Aug 18;2016:2093171. doi: 10.1155/2016/2093171 (PMC5007364; doi:10.1155/2016/2093171)
Supplement: Supplementary file 1 — Figure S1 Process of animal experiment: The SD rats were induced IGT and T2DM by high-fat-high-sugar chow and low dose streptozocin injection. Diabetic rats were then treated with sitagliptin. Feces were collected at four points in the process, representing normal control, obesity, diabetes and sitagliptin-treated condition respectively. [file 2093171.f1.zip › Fig.S3-Shannon Curve.docx]

Obe9 NC6

5

C7_4

Sit9 SNit5C4NC2

SSiti6tO2be1

Sit10

Sit4

Sit5

Sit3

Obe7

Obe5

NC9

NC1

NC8

NC7

Sit4

DM6

Obe3

4

DMD5_M31OC0b2Oe_b62e10 DMN8C3

DM4

NC10

Obe8

NC5

Obe4

DM7 DM9 DM3

DM2DM1

**Rarefaction Measure:r_shannon**

3

2

1

0

0 2000 4000 6000 8000

**Number of Reads Sampled**

label: 0.97

Fig.S3 Shannon-rarefaction Curve.Diversity estimator Shannon of each sample amount of sequencing in different depth of sequencing, the sequencing data is enough to reflect the vast majority of microbes information in the sample as the curve tends to flat.
