# Supplementary material for: Microflora Disturbance during Progression of Glucose Intolerance and Effect of Sitagliptin: An Animal Study
Source: J Diabetes Res. 2016 Aug 18;2016:2093171. doi: 10.1155/2016/2093171 (PMC5007364; doi:10.1155/2016/2093171)
Supplement: Supplementary file 1 — Figure S1 Process of animal experiment: The SD rats were induced IGT and T2DM by high-fat-high-sugar chow and low dose streptozocin injection. Diabetic rats were then treated with sitagliptin. Feces were collected at four points in the process, representing normal control, obesity, diabetes and sitagliptin-treated condition respectively. [file 2093171.f1.zip › Fig.S4-heatmap.docx]

Bifidobacterium Adlercreutzia Peptococcus Butyricimonas Treponema 4C0d−2_norank Escherichia−Shigella Christensenella

Erysipelotrichaceae_Incertae_Sedis Parabacteroides

Desulfovibrio Parasutterella Phascolarctobacterium Ruminococcus RF9_norank

Ruminococcaceae_Incertae_Sedis Roseburia

Oscillibacter Subdoligranulum Shuttleworthia vadinBB60_norank Flavonifractor Anaerofustis Acetitomaculum Veillonella Paenibacillus Brevundimonas Actinomyces Marvinbryantia Coprococcus Coprobacillus Streptococcus

Clostridiales_Incertae_Sedis Lactococcus

Helicobacter Candidate_division_TM7_norank Anaerotruncus

Alistipes ratAN060301C_norank Anaerostipes Oceanobacillus Barnesiella

Rikenella Enterorhabdus Carnobacterium Thalassospira Papillibacter RC9_gut_group Odoribacter Butyrivibrio Turicibacter Clostridium

Peptostreptococcaceae_Incertae_Sedis Bacillus Lachnospiraceae_Incertae_Sedis Blautia

Prevotella Bacteroides Allobaculum Unclassified S24−7_norank uncultured Lactobacillus Lysinibacillus Collinsella Staphylococcus

Candidatus_Arthromitus Brochothrix Lachnospira Anaerovorax Oscillospira Anaerosporobacter Holdemania

Rothia Corynebacterium RF3_norank Caldicoprobacter

Candidate_division_OP3_norank Candidate_division_OD1_norank Pelomonas

Curvibacter Planococcaceae_Incertae_Sedis Chryseobacterium

Leuconostoc Flavobacterium Enterococcus Erysipelotrichaceae_norank Propionibacterium Gordonibacter Stenotrophomonas Rhodobium Subgroup_6_norank

NC1 NC2 NC3 NC4 NC5 NC6 NC7 NC8 NC9 NC10

Obe1 Obe2 Obe3 Obe4 Obe5 Obe6 Obe7 Obe8 Obe9 Obe10 DM1 DM2 DM3 DM4 DM5 DM6 DM7 DM8 DM9 DM10

Sit1 Sit2 Sit3 Sit4 Sit5 Sit6 Sit7 Sit8 Sit9 Sit10

# 0 0.02 0.26 3.71 55.92

Relative abundance of community (%)

Fig.3 Bacterial distribution among samples. The bacterial phylogenetic tree was calculated using the neighbor-joining method and the relationship among samples was determined by Bray distance and the complete clustering methodology. The heatmap plot depicts the relative abundance of each bacterial genus (variables clustering on the Y-axis) within each sample (X-axis clustering). The relative values for bacterial family are depicted by color intensity with the legend indicated at the bottom of the figure. Clusters based on the distance of the samples along the X-axis and the bacterial genus along the Y-axis are indicated in the upper and left of the figure, respectively.
