# Supplementary material for: Microflora Disturbance during Progression of Glucose Intolerance and Effect of Sitagliptin: An Animal Study
Source: J Diabetes Res. 2016 Aug 18;2016:2093171. doi: 10.1155/2016/2093171 (PMC5007364; doi:10.1155/2016/2093171)
Supplement: Supplementary file 1 — Figure S1 Process of animal experiment: The SD rats were induced IGT and T2DM by high-fat-high-sugar chow and low dose streptozocin injection. Diabetic rats were then treated with sitagliptin. Feces were collected at four points in the process, representing normal control, obesity, diabetes and sitagliptin-treated condition respectively. [file 2093171.f1.zip › Fig.S5 lefse_LDA.docx]

Title: Microflora disturbance during progression of glucose intolerance and effect of Sitagliptin: an animal study

Author: Xinfeng Yan, Bo Feng, Peicheng Li, Zhaosheng Tang, Lin Wang

NC Obesity DM Sit

Ruminococcaceae Erysipelotrichales Erysipelotrichaceae Erysipelotrichia

Other uncultured Bacteroidaceae Bacteroides Oscillibacter Proteobacteria

Other Deltaproteobacteria Desulfovibrionaceae Desulfovibrionales Desulfovibrio Curvibacter Betaproteobacteria Burkholderiales Porphyromonadaceae Alcaligenaceae Parasutterella Parabacteroides Actinobacteria

Family_XIII_Incertae_Sedis

Coriobacteriales Coriobacteriia Coriobacteriaceae Peptococcaceae Rhodocyclaceae Rhodocyclales uncultured Comamonadaceae Spirochaetae Spirochaetaceae

Treponema Spirochaetales Spirochaetes uncultured uncultured Bifidobacteriales Bifidobacterium Peptococcus Bifidobacteriaceae

Other Other Adlercreutzia Actinobacteria uncultured

Gammaproteobacteria Enterobacteriaceae Enterobacteriales Escherichia_Shigella

Blautia Bacillales Bacillaceae

Bacillus Coprococcus uncultured Christensenellaceae

Collinsella Marvinbryantia Streptococcaceae Coprobacillus Lactococcus Peptostreptococcaceae

uncultured Clostridiaceae Clostridium Turicibacter

Other Incertae_Sedis Tenericutes Mollicutes norank

RF9

norank Staphylococcaceae Staphylococcus Carnobacterium Carnobacteriaceae Streptococcus

Bacilli Lactobacillales Lactobacillaceae Lactobacillus Prevotellaceae uncultured Ruminococcus Rikenellaceae

Alistipes Anaerotruncus Anaerostipes Barnesiella

norank norank Candidate_division_TM7

norank norank RC9_gut_group Odoribacter Butyrivibrio Rhodospirillales Thalassospira Rhodospirillaceae

Listeriaceae Brochothrix

Other Other

# 0 1 2 3 4 5 6

LDA SCORE (log 10)
